# Effects of physical activity on depressive symptoms in middle-aged and younger-old adults: a systematic review and meta-analysis

**DOI:** 10.3389/fpsyg.2026.1817598

**Published:** 2026-07-01

**Authors:** Fan Xia, Sujie Mao, Jingfeng Wang, Bo Chen, Ying Li, Mi Zheng

**Affiliations:** 1 Graduate School, Harbin Sport University, Harbin, China; 2 School of Traditional Chinese Medicine, Chongqing Three Gorges Medical College, Chongqing, China; 3 Department of Exercise Physiology, Beijing Sport University, Beijing, China

**Keywords:** depression, depressive disorder, exercise, middle-aged and younger-old adults, physical activity

## Abstract

**Background:**

Depression is a common mental health issue in middle-aged and younger-old adults. It can substantially impair their well-being and daily functioning. Physical activity is often recommended to help with depression, but its effectiveness for middle-aged and younger-old adults is not clear. The most effective exercise prescription also remains to be determined. This systematic review aims to answer two questions: Does physical activity reduce depressive symptoms in this group? And if so, which workout parameters work better?

**Methods:**

PubMed, CNKI, Wanfang Data, Embase, VIP, and the Cochrane Library were searched for randomized controlled trials (RCTs) to examine the impact of physical activity on this population. The search timeframe was based on the introduction of databases up to May 15, 2025. The risk of bias was assessed in each included study using the Cochrane Handbook. All meta-analyses were conducted with Review Manager 5.4.

**Results:**

Seven studies (9 RCTs, 494 participants) were included. Physical activity reduced depressive symptoms in medication-free participants. However, in the few studies that examined exercise as an adjunct to stable antidepressant treatment, no additional benefit was observed. In subgroup analyses, Tai Chi was associated with an effect estimate that differed from those of walking or running. Interventions with total duration >6 months, session length ≥60 min, and frequency ≥3 times per week showed greater reductions in depressive symptoms compared with those below these thresholds.

**Conclusion:**

Because all subgroup comparisons were indirect and no direct head-to-head trials were included, no conclusion about the superiority of any exercise type or specific dose parameter can be drawn. Preliminary evidence suggests that for medication-free participants, long-term Tai Chi practice (≥3 times/week, >60 min/session, ≥6 months) was associated with a greater reduction in depressive symptoms in exploratory subgroup analyses. Given the high heterogeneity and other methodological limitations noted, further research with larger samples and more rigorous designs is required before definitive conclusions can be drawn.

**Systematic review registration:**

https://www.crd.york.ac.uk/PROSPERO/view/CRD420251125076, identifier CRD420251125076.

## 1 Introduction

Depression is now recognized as a common mental disorder worldwide (World Health Organization, 2018). It is characterized by low mood, sadness, irritability, and poor sleep quality. It increases the risk of cardiovascular disease mortality by 2–3 times. It may also trigger respiratory system diseases and cancer. Furthermore, depression is significantly positively correlated with all-cause mortality (Wang et al., 2024). It is a risk factor for healthy life expectancy (Lu et al., 2021). Epidemiological surveys show that over 350 million people suffer from depression worldwide. This disease has become the mental disorder with the greatest economic burden (Lu et al., 2021). Middle-aged and younger-old adults are at high risk for depression due to factors such as disease susceptibility and social role transitions. The prevalence rate in this population is approximately 31.9% (Wang C. et al., 2025).

Given the increasing prevalence of depression and its serious consequences, effective interventions are urgently needed to improve healthy life expectancy in this population. Clinical evidence shows limitations of pharmacotherapy and psychotherapy for this population. Pharmacotherapy may cause side effects such as obesity, tremors, sleep disturbances, and anxiety (Wang et al., 2022). These side effects reduce patient adherence. Long-term drug use may lead to drug accumulation and increase cardiovascular risk. It can also induce serious reactions, including serotonin syndrome and syndrome of inappropriate antidiuretic hormone secretion (Joseph et al., 2024), and raise fall risk. Psychotherapy can reduce drug dependence, but its efficacy is limited. A meta-analysis of diabetic patients with depression found that only 17% achieved remission through psychotherapy alone (Mather et al., 2022).

Furthermore, a 2018 World Health Organization (WHO) report stated that fewer than half of people with depression worldwide receive effective treatment. In many countries, this figure drops below 10% (World Health Organization, 2018). Major barriers include lack of resources, insufficient trained providers, and stigma around mental disorders. This treatment gap may be more prominent in the middle-aged and younger-old adults with depression. This is because this group tends to delay seeking medical care, and their depressive symptoms are easily mistaken for a part of the normal aging process, which in turn leads to lower treatment coverage. Given these limitations, safe, appropriate, and more cost-effective interventions are urgently needed.

Clinical guidelines in multiple countries recommend physical activity as a comprehensive management approach to promote physical and mental health (Veronese et al., 2024). It may not only partially replace or assist medication and psychological therapy (Singh et al., 2023), but also indirectly alleviate depression by improving cognitive function and motor ability (Gisina and Yarygin, 2025). However, these studies (Singh et al., 2023; Veronese et al., 2024; Gisina and Yarygin, 2025) did not clearly propose specific exercise prescriptions for this population, such as the appropriate type, frequency, and duration of activity. They also did not distinguish the intervention effects of different exercise types. Furthermore, the existing meta-analysis on this topic leave considerable room for improvement (Zhang et al., 2025).

Existing studies failed to distinguish whether exercise was used as an independent intervention or combined with antidepressant medication, and the confounding effect of concomitant antidepressant use was not extracted. In addition, previous studies included participants with comorbid conditions such as hypertension, cancer, or stroke, which may confound the specific effect of physical activity. Likewise, those studies lacked in depth discussion of the mechanisms linking exercise to effects on this population.

To address these gaps, this meta-analysis extends prior work in four ways. It stratifies effects by antidepressant medication status to isolate the independent effect of exercise. Next, it reports prediction intervals and GRADE certainty, providing a more transparent assessment of the heterogeneity inherent in exercise trials. It adopts a hypothesis generating interpretation of subgroup findings, recognizing the exploratory nature of dose related analyses. And it strictly excludes participants with major comorbidities to reduce confounding.

Therefore, although many studies have confirmed that physical activity improves depression, it remains unclear whether this effect differs depending on patients’ use of antidepressants. No widely accepted exercise intervention has yet been established for middle aged and younger old adults with depression. Although some randomized controlled trials have proposed specific recommendations (Xie, 2011), these recommendations vary across studies and have not been integrated by meta-analysis systems. This meta-analysis aims to synthesize the available evidence, identify potentially effective exercise parameters, and discuss potential mechanisms based on previously published experiments, thereby providing a clearer, evidence-based framework for prescription.

## 2 Materials and methods

The design of this study was conducted in accordance with the Cochrane Handbook for Systematic Reviews of Interventions. To ensure clear and complete reporting, we adhered to the PRISMA 2020 checklist. This review and meta-analysis set out to examine how physical activity affects depression in middle-aged and younger-old adults. The protocol was registered prospectively with the International Prospective Register of Systematic Reviews (PROSPERO) and is available under the number CRD420251125076.

### 2.1 Inclusion criteria

This systematic review was guided by the PICOS framework (Participants, Interventions, Comparisons, Outcomes, Study Design) as recommended in the Cochrane Handbook. The specific criteria were as follows:

(1) Participants: The study included middle-aged and younger-old adults with depression diagnosed using depression scales, with a mean age between 45 and 65 years. Throughout this manuscript, the term “younger-old adults” is used as an operational inclusion criterion for this systematic review. We acknowledge that this term is not a standard age classification in the international literature (World Health Organization, 2018). It was adopted solely to distinguish adults aged 45–65 years from the conventionally defined “older adults” (≥65 years). This range was chosen to minimize heterogeneity from advanced age-related comorbidities (e.g., frailty, multimorbidity) that could confound the specific effect of physical activity, consistent with prior systematic reviews in this area (Richmond DiBello et al., 2024; Sanchez et al., 2024). No restrictions were placed on nationality, sex, ethnicity, or severity of depression.
(2) Interventions: All forms and durations of exercise interventions were included.
(3) Comparators: The control group received usual care only and did not participate in any exercise intervention.
(4) Outcomes: The primary outcome measures were:

- Beck’s Depression Inventory (BDI).
- Hamilton Depression Scale (HAMD; or Hamilton Rating Scale for Depression, HRSD).
- Geriatric Depression Scale (GDS).

(5) Study Design: Randomized controlled trials (RCTs) published in English or Chinese.

### 2.2 Exclusion criteria

(1) Non-English or non-Chinese full-text RCT articles.
(2) No reported outcome measures.
(3) Duplicate publications.
(4) Missing continuous variable data for key statistics (e.g., mean, standard deviations).
(5) Studies with incomplete experimental design or animal experiments were excluded.
(6) Mean age not within 45–65 years.
(7) Other diseases including cancer, Alzheimer’s disease, postpartum depression, Parkinson’s disease, stroke and others.

### 2.3 Literature search strategy

The literature search strategy was customized according to the syntax and controlled vocabulary of each database(PubMed, CNKI, Wanfang Data, Embase, VIP, Web of Science, EBSCO, and the Cochrane Library). The search covered all records from the inception of each database up to May 15, 2025. A comprehensive search strategy was adopted to maximize the scope of keywords, ensuring that the results focused on physical activity interventions for depression in middle-aged and younger-old adults. In Chinese databases, the search terms included “exercise,” “physical activity,” “depression,” “middle-aged and younger-old adults,” and “randomized controlled trial.” The English search terms employed were “exercise,” “sport,” “physical activity,” “middle-aged and younger-old adults,” “depression,” “depressive disorder,” “randomized controlled trial,” and “RCT.” The complete search strings for all databases are presented in Table 1.

**Table 1:** Search strategy.

Database	Number	Search strategy
PubMed	#1	Exercise [Mesh] OR Physical Activity [Title/Abstract] OR Training [Title/Abstract] OR Action [Title/Abstract] OR Functional training [Title/Abstract] OR Sport [Title/Abstract]
	#2	Frail elderly [Mesh] OR Aged [Mesh] OR Elder [Title/Abstract] OR Senior [Title/Abstract] OR Older people [Title/Abstract] OR Older adults [Title/Abstract]
	#3	Depression [Mesh] OR Depressive disorder [Mesh] OR Mental disorders [Title/Abstract] OR Anxiety [Title/Abstract] OR Depressive syndrome [Title/Abstract]
	#4	Randomized controlled trial [Publication Type] OR Clinical trial [Publication Type] OR Controlled clinical trial [Publication Type] OR Randomized [Title/Abstract]
	#5	#1 AND #2AND #3 AND #4
Embase	#1	Exercise/exp. OR Physical activity: ab,ti OR Training: ab,ti OR Functional training: ab,ti OR Sport: ab,ti
	#2	Frail elderly /exp. OR Aged/exp. OR Elder: ab,ti OR Senior: ab,ti OR Older adult: ab,ti OR Older people: ab,ti
	#3	Depression/exp. OR Depressive disorder/exp. OR Mental disorders: ab,ti OR Anxiety: ab,ti OR Depressive syndrome: ab,ti
	#4	Randomized controlled trial/exp. OR Clinical trial/exp. OR Controlled clinical trial/exp. OR Randomized: ab,ti
	#5	#1 AND #2AND #3 AND #4
Cochrane library	#1	Exercise
	#2	Exercise: ab,ti, kw OR Physical Activity: ab,ti, kw OR Activities, Physical: ab,ti, kw OR Training: ab,ti, kw OR Functional training: ab,ti, kw OR Sport: ab,ti,kw
	#3	#1OR #2
	#4	Depression
	#5	Depressive syndrome: ab,ti, kw OR Depressive disorder: ab,ti,kw OR OR Mental Depression: ab,ti,kw OR Anxiety: ab,ti,kw
	#6	#4OR #5
	#7	Frail Elderly: ab,ti,kw OR Aged ab,ti,kw OR Elder: ab, ti OR Senior: ab, ti OR Older adults: ab,ti OR Older people: ab,ti
	#8	Randomized controlled trial ab,ti, kw OR Randomized: ab,ti, kw OR Clinical trial ab,ti, kw OR Controlled clinical trial: ab,ti, kw
	#9	#3 AND #6AND #7 AND #8
Web of science	#1	TS=Depression OR Depressive syndrome OR Depressive disorder OR Mental Depression OR Anxiety
	#2	TS=Frail Elderly OR Aged OR Elder OR Senior OR Older people OR Older adults
	#3	TS = Exercise OR Physical Activity OR Training OR Action OR Functional training OR Sport
	#4	TS = Randomized controlled trial OR Clinical trial OR Controlled clinical trial OR Randomized OR DT = Randomized Controlled Trial OR Clinical Trial
	#5	#1 AND #2AND #3 AND #4
EBSCO	#1	DE Exercise OR TI Physical activity OR AB Physical activity OR TI training OR AB training TI Action OR AB Action OR TI Functional training OR AB Functional training OR TI Sport OR AB Sport
	#2	DE Aged OR TI Elder OR AB Elder OR TI Older adult OR AB Older adult TI Older people OR AB Older people
	#3	DE Depression OR TI Depressive syndrome OR AB Depressive syndrome OR TI anxiety OR AB anxiety TI Mental Depression OR AB Mental Depression TI Depressive disorder OR AB Depressive disorder
	#4	TI Randomized controlled trial OR AB randomized controlled trial OR TI Clinical trial OR AB Clinical trial OR TI Randomized OR AB Randomized
	#5	#1 AND #2AND #3 AND #4
CNKI	#1	SU=Depression OR Depressive syndrome OR Mental Depression
	#2	SU=Frail Elderly OR Aged OR Elder OR Older people
	#3	SU = Exercise OR Physical Activity OR Training OR Functional training
	#4	SU = Randomized controlled trial OR Clinical trial
	#5	#1 AND #2AND #3 AND #4
Wanfang Data	#1	Topic = Depression OR Depressive syndrome OR Mental Depression
	#2	Topic = Frail Elderly OR Aged OR Elder OR Older people
	#3	Topic = Exercise OR Physical Activity OR Training OR Functional training
	#4	Topic ==Randomized controlled trial OR Clinical trial
	#5	#1 AND #2AND #3 AND #4
VIP	#1	M = Depression OR Depressive syndrome OR Mental Depression
	#2	M = Frail Elderly OR Aged OR Elder OR Older people
	#3	M = Exercise OR Physical Activity OR Training OR Functional training
	#4	M = Randomized controlled trial OR Clinical trial
	#5	#1 AND #2AND #3 AND #4

### 2.4 Literature screening and data extraction

The PRISMA guidelines were followed for this process. Two reviewers, FX and JW, independently screened the records, extracted the data, and assessed the methodological quality of the studies based on the predefined eligibility criteria. Any disagreements were resolved through consultation with Sujie Mao. The screening process consisted of two stages. Initially, the reviewers screened the titles, excluding clearly irrelevant articles at this stage. Subsequently, they independently assessed the abstracts and full texts of the remaining records to determine the final studies for inclusion.

We systematically collected the following key characteristics from each eligible study: (1) Basic information (authorship, publication year, country of origin, participant demographics); (2) Key elements for risk of bias assessment; (3) Details of the intervention; (4) For outcome measures, means and standard deviations were obtained directly from the text when possible. If they were not available, we calculated them from other reported statistics, including minimum and maximum values, or the median and quartiles. When needed, the Web Plot Digitizer was used to extract data from figures (Shi et al., 2020; Liu and An, 2021).

### 2.5 Risk assessment of literature bias

Following the Cochrane Handbook guidelines (Higgins et al., 2024), Xia and Wang independently evaluated the methodological quality of all included trials. Risk of bias was assessed across key domains: selection bias (random sequence generation and allocation concealment), performance bias, detection bias, attrition bias, reporting bias, and other potential sources of bias.

### 2.6 Statistical analysis

Literature screening and management were handled using Zotero. For data extraction, Excel was relied upon to maintain analytical reliability. Heterogeneity testing, meta-analysis, and forest plot generation were carried out in Review Manager 5.4. In this study, all outcome measures were continuous variables, but they were measured in different units. To address this, standardized mean difference (SMD) with 95% confidence intervals (CIs) was used as the effect size. The I^2^ statistic served as the primary tool for quantifying heterogeneity across studies.

When heterogeneity was low (I^2^ ≤ 50%), a fixed-effect model was chosen. In cases with I^2^ > 50%, indicating substantial heterogeneity, a random-effects model was opted for. Where notable heterogeneity emerged, its potential sources were explored. After excluding obvious clinical heterogeneity, meta-analysis proceeded using the random-effects model. To address significant clinical heterogeneity, either subgroup analyses or sensitivity analyses were performed by sequentially omitting individual studies.

The assessment was performed following the Cochrane Handbook and GRADE guidelines, using the GRADE pro GDT tool. The overall certainty of evidence for the primary outcome was assessed using the GRADE (Grading of Recommendations, Assessment, Development and Evaluations) framework, considering risk of bias, inconsistency, indirectness, imprecision, and publication bias.

## 3 Results

### 3.1 Literature search and selection

**Figure 1:**
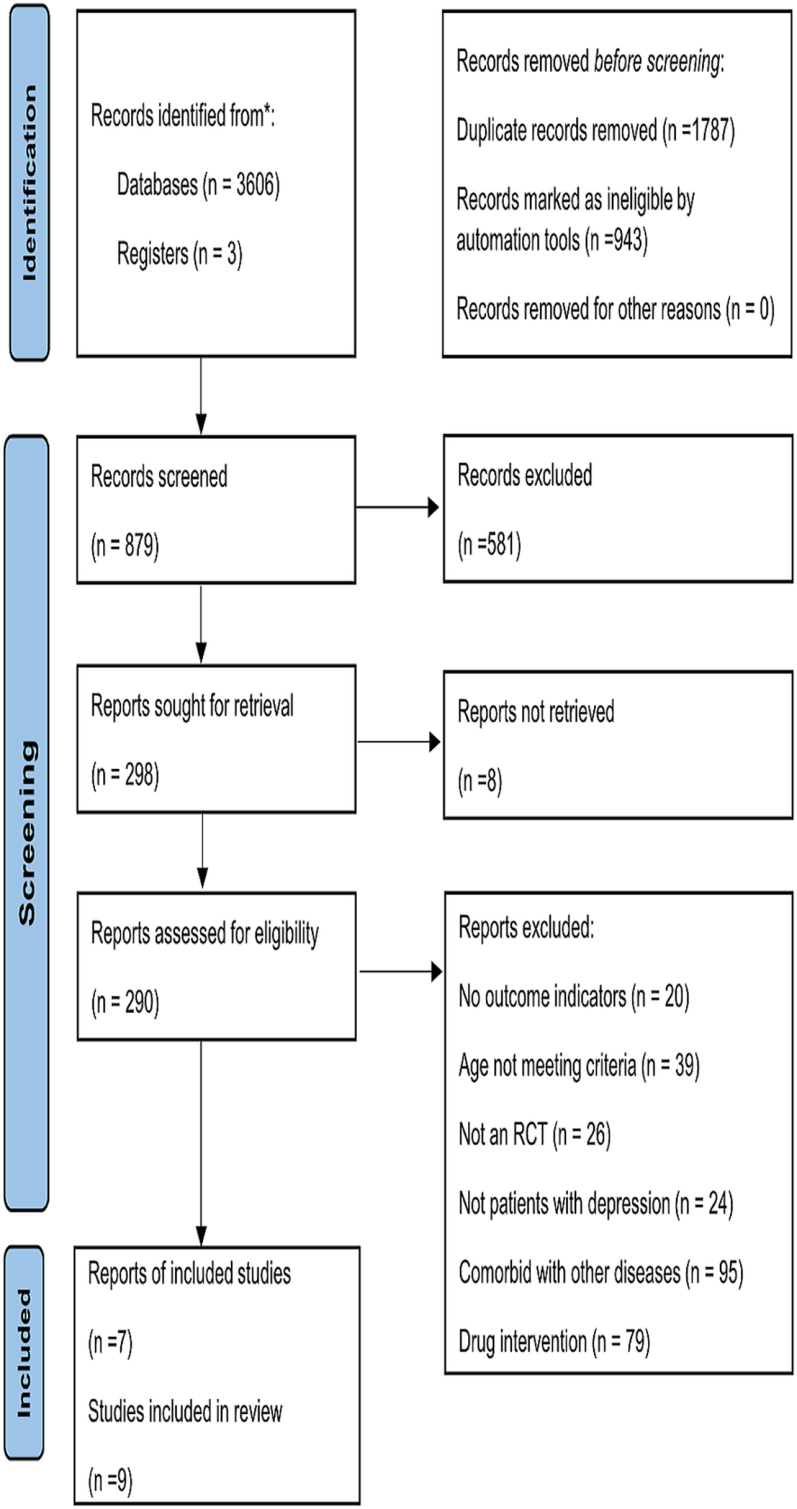
Literature search process and results. * The databases searched and the number of records retrieved were: CNKI (*n* = 703), Wan Fang Data (*n* = 295), VIP (*n* = 126), Web of Science (*n* = 208), EBSCO (*n* = 110), PubMed (*n* = 1872), The Cochrane Library (*n* = 221), and Embase (*n* = 71). After deduplication, title/abstract screening, full-text assessment, and application of inclusion/exclusion criteria, seven studies (comprising nine randomized controlled trials) were finally included.

The initial database search yielded 3,909 records. After removing duplicate entries, he titles and abstracts were screened. This screening was guided by predefined inclusion and exclusion criteria, ultimately identifying seven eligible articles (Blumenthal et al., 1999, 2007; Xie, 2011; Afonso et al., 2012; Zhao et al., 2015; Rao et al., 2021; Yu et al., 2023). These comprised four English and three Chinese studies, encompassing nine RCTs. The complete screening process is outlined in Figure 1.

### 3.2 Characteristics of included studies

We included seven articles in this review, which covered nine randomized controlled trials. In total, 494 participants took part in these studies. Their ages ranged from 45 to 65 years. Exercise interventions in the experimental groups included Tai Chi, yoga, and running/walking. For the control groups, the participants received no exercise intervention. The included studies exhibited considerable heterogeneity in intervention protocols. Specifically, intervention durations ranged from 2 to 12 months. Sessions were held 3 to 8 times per week, with each session lasting between 30 and 150 min. Table 2 presents the general characteristics of the included studies (Blumenthal et al., 1999, 2007; Xie, 2011; Afonso et al., 2012; Zhao et al., 2015; Rao et al., 2021; Yu et al., 2023).

**Table 2:** Basic characteristics of the included literature.

Study	Country	Sample size T/C	Age (years)		Sex (M/F)		Interventions		Duration (months)	Outcome indicator
			T	C	T	C	T	C		
Xie (2011)	China	30/30	58.90 ± 4.28	59.7 ± 3.54	15/15	15/15	Only Tai Chi; 3×/week, 60 min/session.	No	6	③
Zhao et al. (2015)	China	26/26	59.2	60.23	12/14	11/15	Only Tai Chi; 3×/week, 30 min/session.	NO	12	③
Rao (2021)	China	15/15	53.91 ± 4.65	53.32 ± 4.99	6/9	5/10	Yes+ Tai Chi; 5×/week, 60 min/session.	Yes	2	②
Blumenthal et al. (1999)	USA	39/41	57 ± 5.8	57 ± 7	10/29	8/33	Yes+ Running/walking; 3×/week, 45 min/session.	Yes	4	②
Blumenthal et al. (2007)	USA	51/49	52 ± 7	52 ± 8	12/39	12/37	Yes+ Supervised running/walking; 3×/week, 45 min/session.	Yes	4	②
Blumenthal et al. (2007)	USA	53/49	53 ± 8	52 ± 8	14/39	12/37	Yes+ Home-based running/walking; 3×/week, 45 min/session.	Yes	4	②
Yu et al. (2023)	China	10/10	60.6 ± 3.1	60.5 ± 7.3	8/2	8/2	Only Moderate running/walking; 3×/week, 150 min/session.	No	3	①
Yu et al. (2023)	China	10/10	59.6 ± 4.6	60.5 ± 7.3	6/4	8/2	Only Vigorous running/walking; 3×/week, 75 min/session.	No	3	①
Afonso et al. (2012)	Brazil	15/15	50–65	50–65	0/15	0/15	Only Yoga; 2×/week, 60 min/session.	No	4	①

T, experimental group; C, control group; NR, not reported. ①, ②, and ③ represent different outcome measures. Detailed definitions are provided in Section 1.1 Inclusion Criteria; Yes: medication; No: non-drug treatment. All included studies had a mean age within the prespecified inclusion range of 45–65 years.

Among the 7 eligible articles, 3 studies explicitly reported that participants were receiving stable antidepressant medication (Blumenthal et al., 1999, 2007; Rao et al., 2021), while the remaining 4 studies did not report medication use.

### 3.3 Included in the risk assessment of bias in the literature

Of the nine RCTs included, six studies specified the randomization techniques they used. The methods included computer-generated random allocation, random number tables, and stratified randomization. The remaining three studies only mentioned randomization without describing the method of sequence generation. Regarding allocation concealment, five studies reported performing it. For the other four studies, this information was unclear. For blinding, five studies reported blinding participants and personnel. The remaining four studies did not provide clear information on blinding of outcome assessment. In terms of outcome data, five studies reported complete data. The other four studies had some missing data, but the reasons for attrition were recorded. None of the nine studies showed signs of other biases.

**Figure 2:**
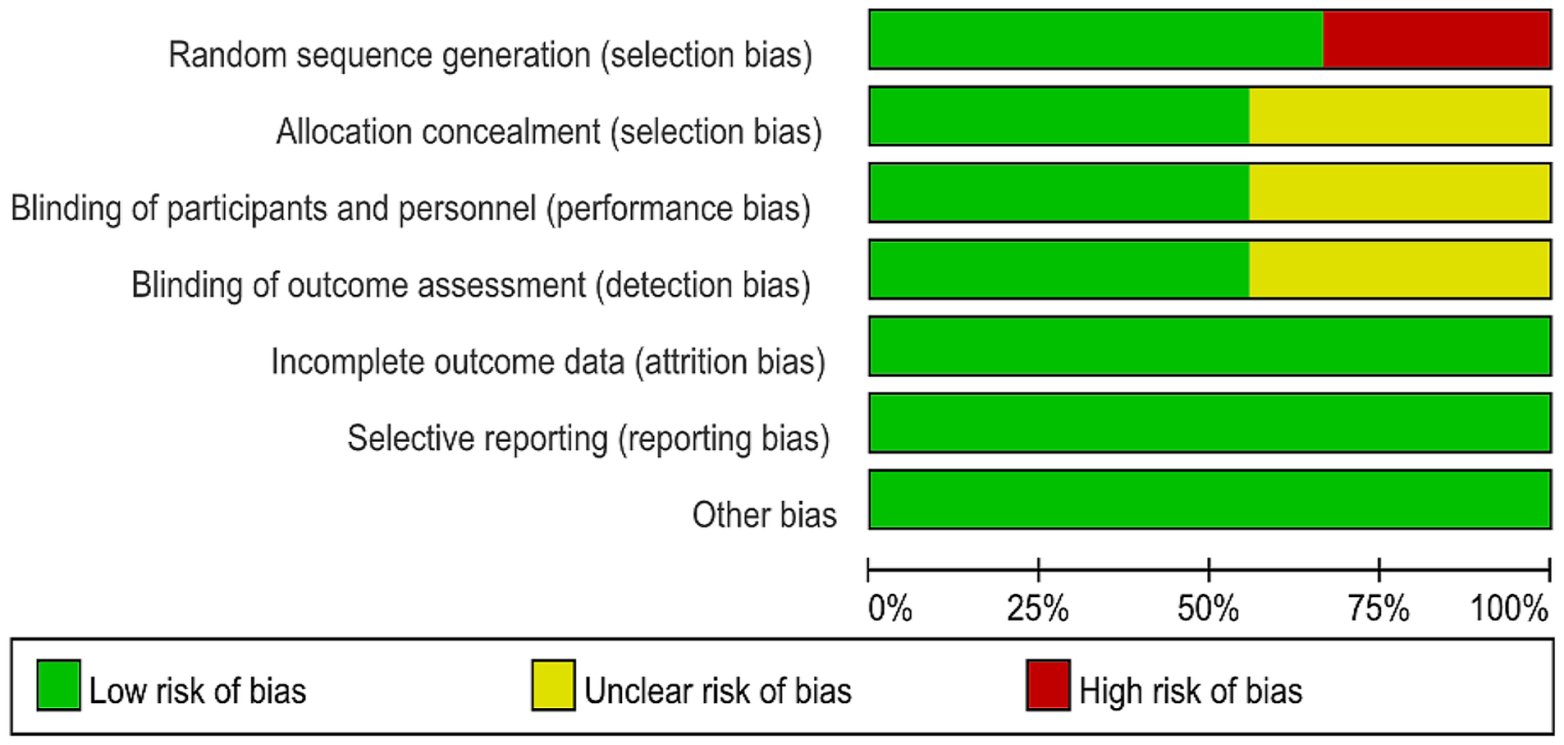
Evaluation of literature bias. Percentage of studies with low, unclear, or high risk across all included studies (*n* = 9 RCTs). The seven domains assessed were: random sequence generation, allocation concealment, blinding of participants and personnel, blinding of outcome assessment, incomplete outcome data, selective reporting, and other bias. Green indicates low risk of bias; yellow indicates unclear risk of bias; red indicates high risk of bias.

**Figure 3:**
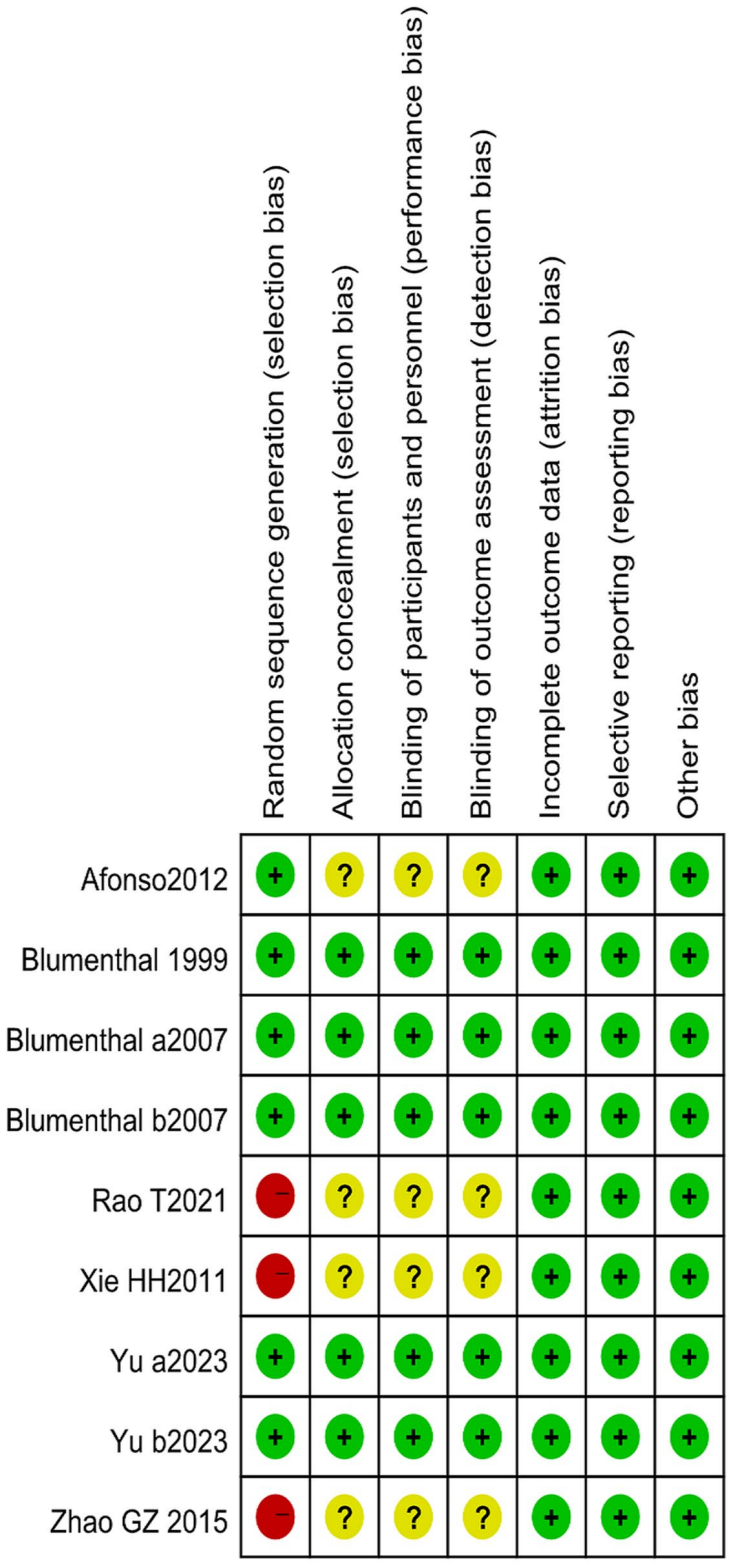
Summary of literature bias. Each row represents one study; each column represents one of the seven domains (as defined in Figure 2). Green (+) indicates low risk; Yellow (?) indicates that the risk is unknown; Red (−) indicates high risk. The color coding and symbols follow the Cochrane Risk of Bias tool.

It is important to note that blinding of participants is inherently unfeasible in exercise trials due to the physical nature of the intervention (Hird et al., 2024). However, blinding of outcome assessors and data analysts is feasible. In our included studies, five reported blinding of outcome assessors, which partially mitigates the risk of detection bias. Nevertheless, the lack of participant blinding may still introduce expectancy or placebo effects, potentially inflating the estimated effect on subjective outcomes. This concern is further addressed in the Limitations section. Overall, the included studies were of moderate methodological quality. The overall risk of bias of the included studies is shown in Figure 2, and the risk of bias for each study is shown in Figure 3.

### 3.4 Overall effect of physical activity intervention on middle-aged and younger-old adults with depression

**Figure 4:**
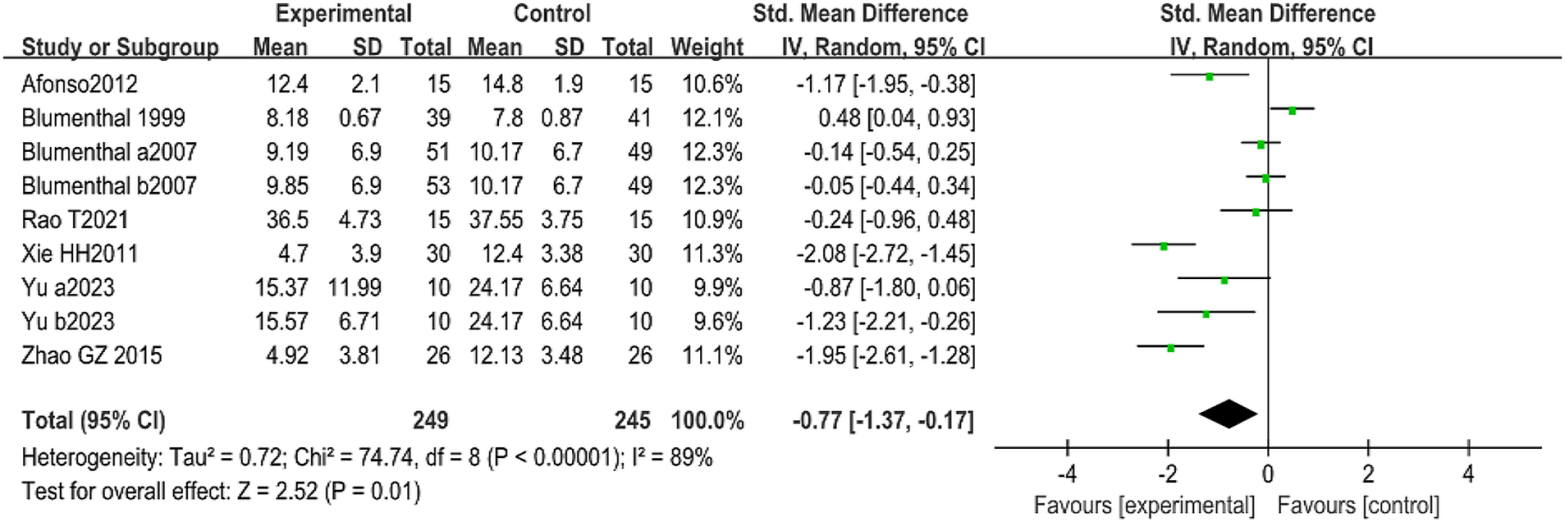
Forest plot of the overall effect of physical activity on depressive symptoms in middle-aged and younger-old adults. Squares represent individual study effect sizes (standardized mean difference, SMD), with square size proportional to the study’s weight in the random-effects model. Horizontal lines represent 95% confidence intervals (CIs). The diamond at the bottom represents the pooled effect estimate; the width of the diamond indicates the 95% CI of the pooled effect. Heterogeneity statistics (τ^2^ = 0.72, I^2^ = 89%) are shown below the forest plot.

A meta-analysis of 9 RCTs was performed. The studies showed significant heterogeneity (*p* < 0.00001, I^2^ = 89%). Therefore, a random-effects model was used. The combined results indicated that physical activity intervention significantly reduced depressive symptoms in middle-aged and younger-old adults (SMD = -0.77, 95% CI: −1.37 to −0.17, *p* = 0.01). Figure 4 displays the forest plot. The random-effects model yielded τ^2^ = 0.72. The 95% prediction interval for the true effect in a future study ranged from −2.90 to 1.36, indicating that while the pooled effect favors exercise, the effect in a new setting could be null or even negative. This prediction interval reflects the substantial heterogeneity across the included studies and does not contradict the statistically significant pooled estimate (Table 3).

**Table 3:** Calculated 95% prediction intervals.

Analysis	k	SMD (95% CI)	τ^2^	95% prediction interval
Overall effect	9	-0.77 (−1.37, −0.17)	0.72	−2.90 to 1.36
Running/walking	5	−0.21 (−0.67, 0.25)	0.18	−1.75 to 1.33
Tai Chi	3	−1.43 (−2.56, −0.31)	0.87	not reported (k < 4)
Duration <6 months	7	−0.34 (−0.76, 0.08)	0.22	−1.67 to 0.99
Duration ≥6 months	2	−2.02 (−2.48, −1.56)	0.00	not reported (k < 4)
Session ≤60 min	7	−0.71 (−1.40, −0.01)	0.79	−3.17 to 1.75
Session >60 min	2	−1.04 (−1.71, −0.37)	0.00	not reported (k < 4)
Frequency ≥3/week	8	−0.73 (−1.37, −0.08)	0.75	−3.00 to 1.54
Medication: Yes	4	0.04 (−0.27, 0.35)	0.04	−1.06 to 1.14
Medication: No	5	−1.54 (−2.01, −1.06)	0.13	−2.92 to −0.16

To investigate the cause of high heterogeneity, sensitivity analysis via the leave-one-out approach was carried out. The combined results showed no significant change in heterogeneity, suggesting that our findings are robust.

#### 3.4.1 Certainty of evidence

The overall certainty was rated low (⊕◯◯◯), downgraded for serious risk of bias (lack of participant blinding), very serious inconsistency (I^2^ = 89, 95% prediction interval crossing zero), and serious imprecision (small sample size, wide confidence interval). This rating does not mean that physical activity is ineffective; rather, it indicates that our confidence in the effect estimate is limited, and future large-scale, well-designed randomized controlled trials are likely to change the estimate. No serious indirectness or publication bias was detected.

### 3.5 Subgroup analysis

To explore whether study-level characteristics affected overall effect estimates, subgroup analyses of key exercise parameters (type, training duration, session duration, frequency) and the effects of concomitant medication were performed among individuals with depression. Reduced heterogeneity within subgroups suggested these parameters contributed to the observed variability.

#### 3.5.1 Exercise type

**Figure 5:**
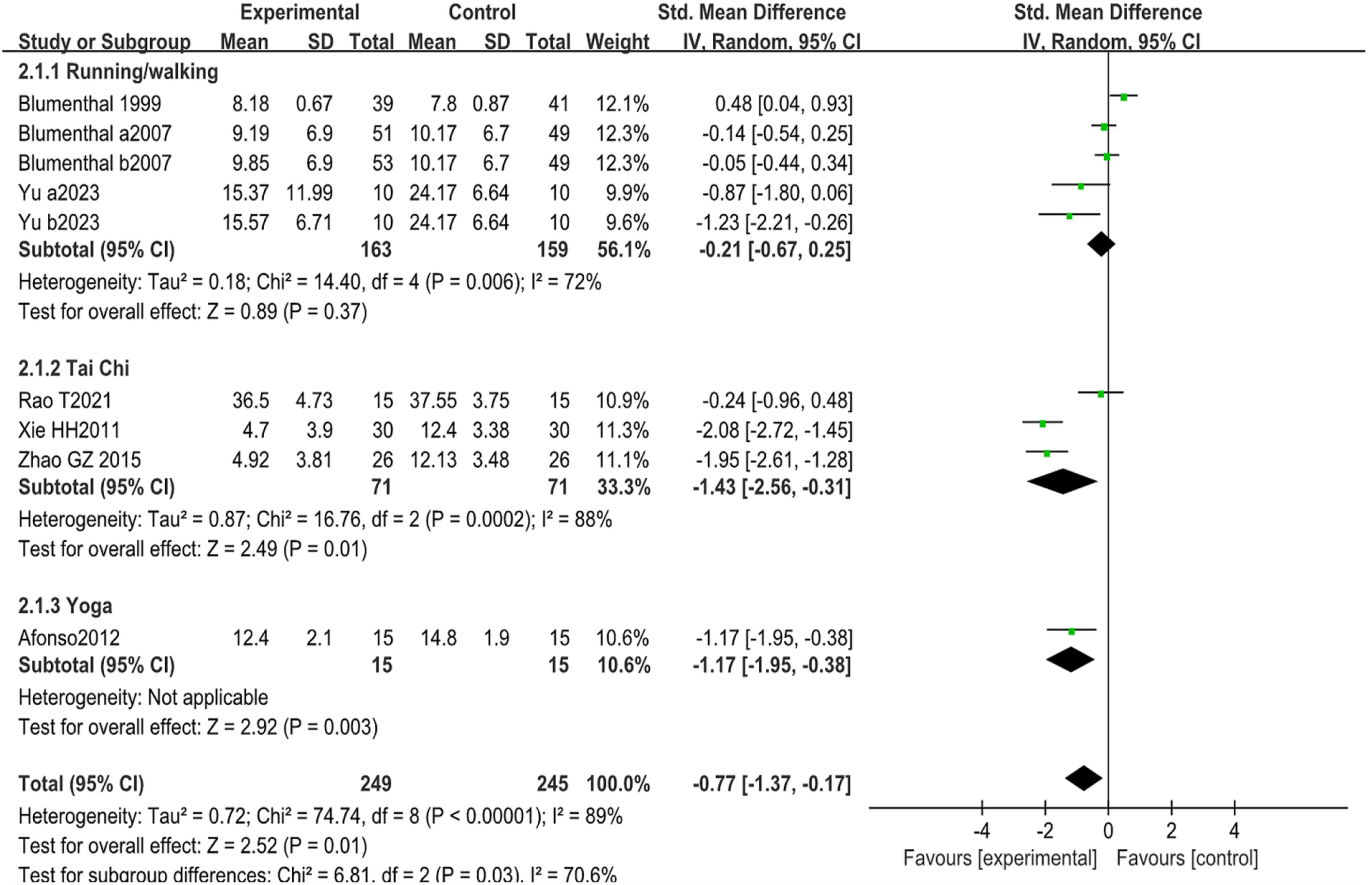
Forest plot of the effect of exercise type on depressive symptoms. Subgroup analyses were performed according to exercise modality: running/walking, Tai Chi, and yoga. The test for subgroup differences is shown at the bottom.

In total, 9 studies involving 494 participants were analyzed. According to the meta-analysis, the type of exercise played a key role in influencing depressive symptoms among middle-aged and younger-old adults. The overall effect was significant (SMD = -0.77, 95% CI [−1.37, −0.17], *p* = 0.01). Further subgroup analyses indicated that larger effect estimates were observed for Tai Chi (SMD = -1.43, 95% CI [−2.56, −0.31], *p* < 0.01), followed by yoga (SMD = -1.17, 95% CI [−1.95, −0.38], *p* = 0.003). In contrast, running/walking did not yield a statistically significant effect (SMD = −0.21, 95% CI [−0.67, 0.25], *p* = 0.37). Figure 5 displays these results. Also, the 95% prediction interval ranged from −1.75 to 1.33, which crosses zero (Table 3). This further indicates that the effect of this exercise modality is uncertain across future studies, consistent with the non-significant pooled estimate.

#### 3.5.2 Training duration

**Figure 6:**
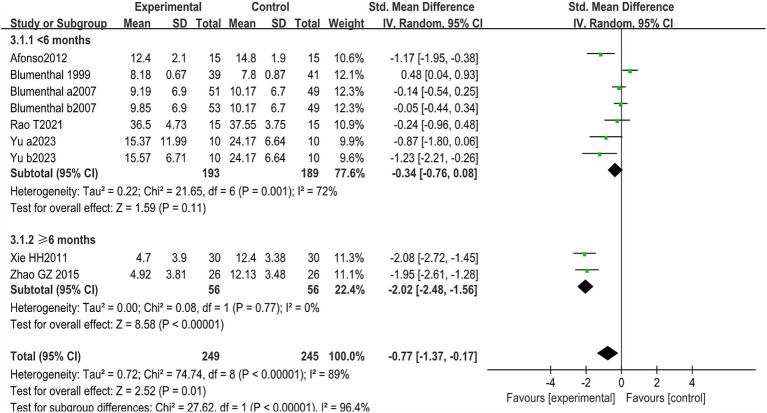
Forest plot of the effect of training duration on depressive symptoms. Subgroup analyses compared interventions lasting <6 months vs. ≥6 months.

The final analysis comprised 9 studies, encompassing 494 participants. Meta-analysis results showed that training duration was a key factor influencing depressive symptoms in middle-aged and elderly patients with depression. The effect was statistically significant [SMD = −0.77, 95% CI (−1.37, −0.17), *p* = 0.01]. Furthermore, subgroup analysis revealed that a training duration of at least 6 months produced significant antidepressant effects. The effect sizes for training duration < 6 months and ≥ 6 months were [SMD = −0.34, 95% CI (−0.76, 0.08), *p* = 0.11] and [SMD = −2.02, 95% CI (−2.48, −1.56), *p* < 0.0001], respectively (Figure 6).

To assess whether the large effect size was driven by a single study, a leave-one-out meta-analysis was performed. After removing Xie, 2011, [SMD = −1.95, 95% CI (−2.61, −1.28)]; after removing Zhao et al. (2015), [SMD = −2.08, 95% CI (−2.72, −1.45)]. Both remained statistically significant and comparable to the original estimate (SMD = −2.02), indicating that the result was not unduly influenced by either individual study. However, the small number of studies limits the generalizability of this finding. Future long-term RCTs are needed to clarify potentially relevant training durations.

#### 3.5.3 Session duration

**Figure 7:**
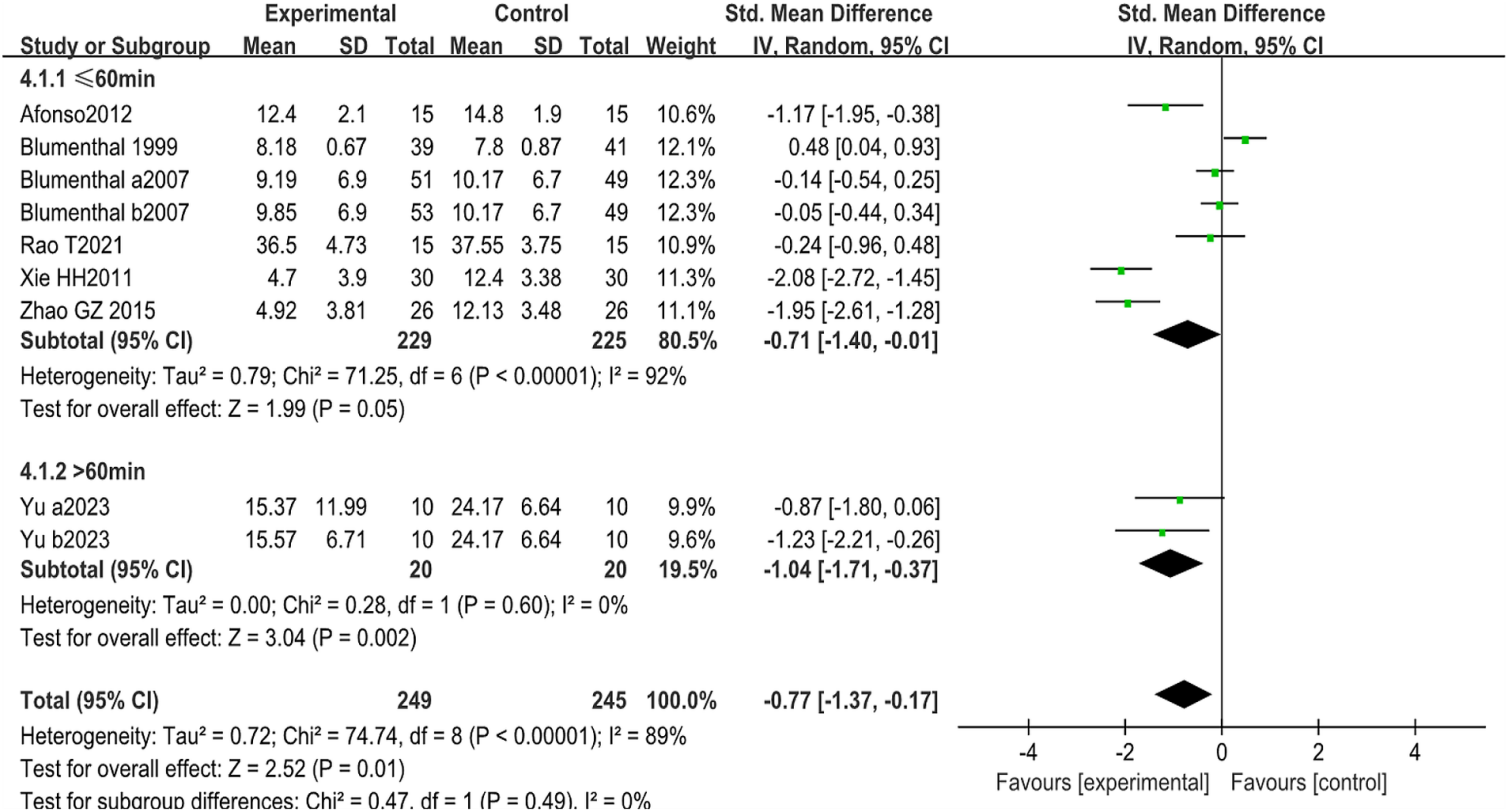
Forest plot of the effect of session duration on depressive symptoms. Subgroup analyses compared session duration ≤60 min vs. >60 min.

A total of 494 participants from 9 studies were included in this review. The meta-analysis showed that session duration had a significant effect on depressive symptoms in this population with depression (SMD = –0.77, 95% CI [−1.37, −0.17], *p* = 0.01). Interventions lasting more than 60 min produced the largest effect size, and this effect remained statistically significant. For sessions of 60 min or less, the effect size was SMD = –0.71 (95% CI [−1.40, −0.01], *p* = 0.05); for sessions longer than 60 min, it was SMD = –1.04 (95% CI [−1.71, −0.37], *p* = 0.002; Figure 7).

#### 3.5.4 Exercise frequency

**Figure 8:**
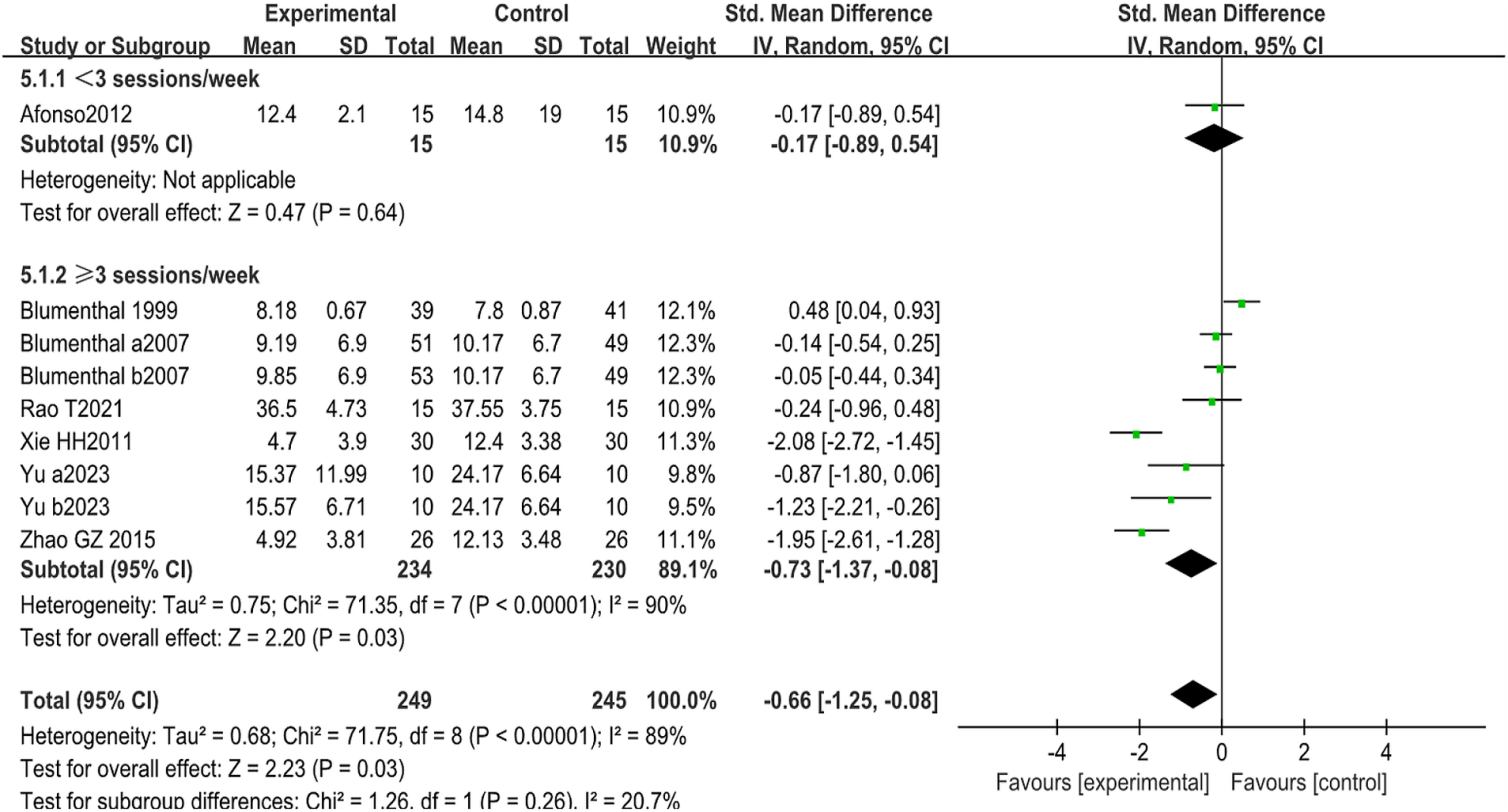
Forest plot of the effect of exercise frequency on depressive symptoms. Subgroup analyses compared exercise frequency <3 sessions/week vs. ≥3 sessions/week.

A total of 494 participants from 9 studies were included in this review. The meta-analysis indicated that exercise frequency had an important influence on this population with depression. The overall effect was significant [SMD = −0.66, 95% CI (−1.25, −0.08), *p* = 0.03]. Further analysis indicated that exercising at least three times per week was associated with depression relief. The effect size for exercise frequency < 3 sessions/week was [SMD = −0.17, 95% CI (−0.89, 0.54), *p* = 0.64]. The effect size for exercise frequency ≥ 3 sessions/week was [SMD = −0.73, 95% CI (−1.37, −0.08), *p* = 0.03] (Figure 8).

#### 3.5.5 Influence of concomitant medication

To examine whether the antidepressant benefits of physical activity are independent of pharmacotherapy, Eligible RCTs were stratified according to participants’ baseline antidepressant use. A total of 494 participants from 9 studies were included in this review. The meta-analysis revealed a significant difference between subgroups (*p* < 0.0001), indicating that the effect of physical activity differs depending on whether participants are already on antidepressants.

**Figure 9:**
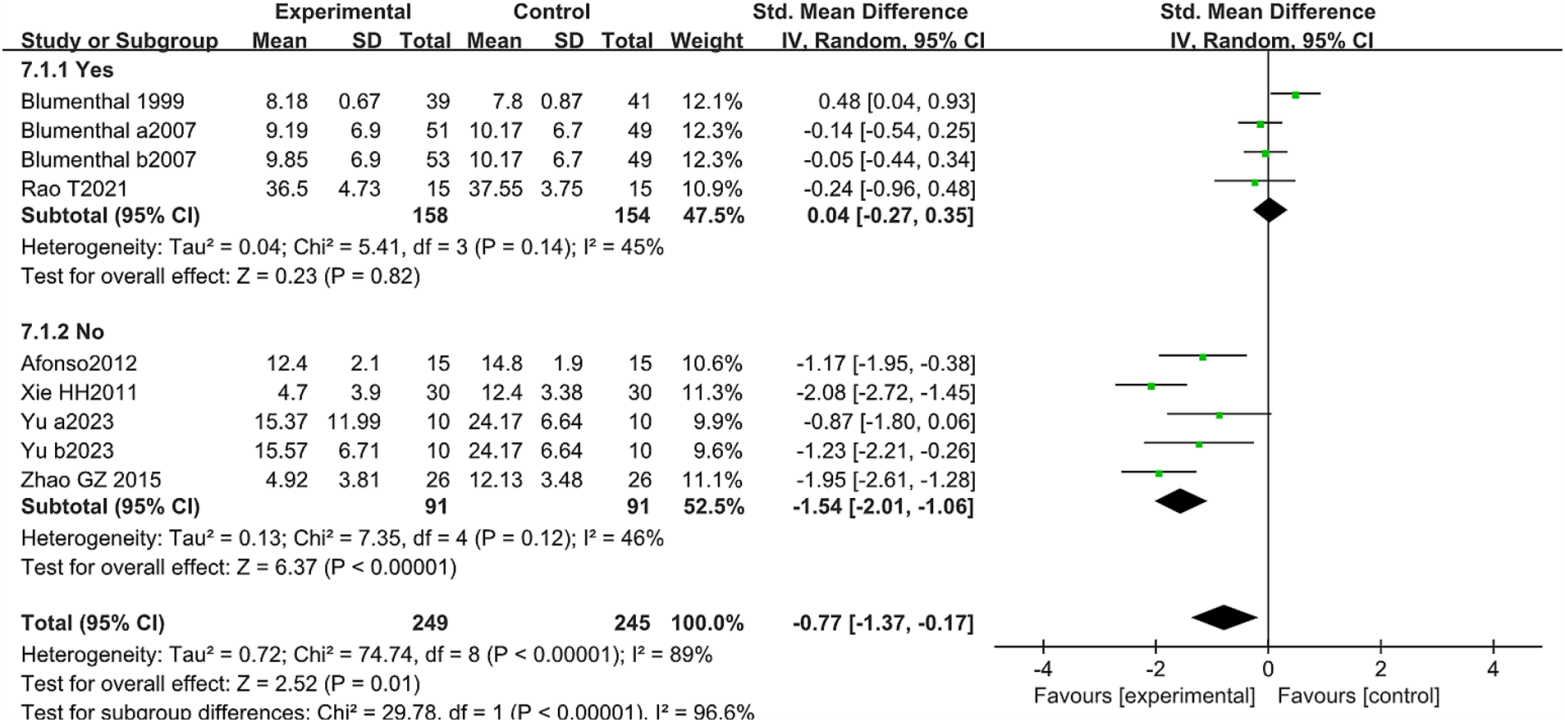
Analysis of combination medications. Subgroup analyses compared participants who were receiving stable antidepressant medication (Yes) vs. medication-free participants (No).

Four RCTs (Blumenthal et al., 1999; Blumenthal et al., 2007; Rao et al., 2021) documented antidepressant treatment across all participants. In these trials, the intervention group received exercise added to medication, whereas controls maintained pharmacotherapy alone. The pooled effect size was non-significant [SMD = 0.04, 95% CI (−0.27, 0.35), *p* = 0.82]. The 95% prediction interval ranged from −1.06 to 1.14, widely crossing zero and consistent with the null pooled effect (Table 3). The remaining five RCTs excluded any pharmacotherapy. In these trials, the intervention group received exercise alone and the control group received no exercise and no medication. The pooled effect was significant [SMD = −1.54, 95% CI (−2.01, −1.06), *p* < 0.0001] (Figure 9). Furthermore, the 95% prediction interval ranged from −2.92 to −0.16, which does not cross zero. This suggests that even in a future study, the effect of physical activity in unmedicated participants is likely to remain beneficial, providing greater confidence in the reproducibility of this finding (Table 3).

## 4 Discussion

The key novel finding of this meta-analysis is that physical activity significantly reduces depressive symptoms only in medication-free middle-aged and younger-old adults, while showing no additional benefit when added to stable antidepressant treatment. Furthermore, in subgroup analyses, Tai Chi showed a larger effect estimate than walking or running. However, because all such comparisons were indirect and potentially confounded by differences in intervention dose, this finding does not support the superiority of Tai Chi over other exercise modalities (Table 4).

**Table 4:** GRADE certainty of evidence for the overall effect of physical activity on depressive symptoms.

Certainty assessment							No of patients		Effect		Certainty	Importance
No of studies	Study design	Risk of bias	Inconsistency	Indirectness	Imprecision	Other considerations	Physical activity	No physical activity	Relative (95% CI)	Absolute (95% CI)		
9	Randomised trials	Serious^a^	Very serious^b^	Not serious	Serious^c^	None	249	245	-	SMD −0.77 SD lower (−1.37 lower to −0.17 lower)	⨁◯◯◯ Very low^a^^,^^b^^,^^c^	CRITICAL

CI, confidence interval; SMD, standardized mean difference.

a Lack of blinding of participants (inherent to exercise trials).

b I^2^ = 89, 95% prediction interval crosses zero (−2.90 to 1.36).

c Total sample size = 494 (< 400), wide 95% CI.

Compared with previous meta-analyses, this study stratified participants by stable antidepressant treatment status. Physical activity relieved depressive symptoms only in medication-free patients and produced no additive benefits when combined with pharmacotherapy. We further computed prediction intervals and GRADE certainty to quantify heterogeneity-derived uncertainty, an approach rarely adopted in earlier reviews. Subgroup results were interpreted exploratorily to propose hypotheses instead of issuing fixed exercise dosage recommendations. Clinically, long-term Tai Chi practice meeting the criteria of no less than three sessions weekly, over 60 min per session and a minimum six-month duration achieves prominent symptom improvement in drug-naive patients. For patients already on stable antidepressants, clinicians should moderate expectations about additional benefits from exercise.

### 4.1 Overall effects

The overall effect size of this meta-analysis confirmed that physical activity effectively alleviates depression in this population. Notably, this result is in line with previous studies by Blumenthal (Blumenthal et al., 2007), Zhang (Zhang et al., 2025), and Rhyner (Rhyner and Watts, 2016). This is also consistent with the WHO recommendations (World Health Organization, 2018). However, our effect size differs from the estimate reported by Rhyner (Rhyner and Watts, 2016). This discrepancy likely reflects heterogeneity in the types of exercise examined. Zhao further showed that sedentary behavior increases depression risk (Zhao et al., 2024). Zhang found that both gentle activities (yoga, Pilates) and more vigorous exercise can benefit depressed older adults (Zhang and Jiang, 2023). Conversely, not all studies have reached consistent conclusions. Several trials suggest that aerobic exercise has only limited effects on depressive symptoms (Yu et al., 2022). These conflicting findings highlight the complexity of the relationship between physical activity and depression in middle-aged and younger-old adults. This relationship clearly warrants further investigation.

Although the meta-analysis showed a statistically significant overall effect of physical activity on depressive symptoms [SMD = --0.77, 95% CI (−1.37, −0.17)], considerable heterogeneity was present (I^2^ = 89%). A pooled estimate with such high heterogeneity should not be interpreted as a single universal effect. Instead, we conducted subgroup analyses to explore potential sources of heterogeneity, including exercise type, intervention duration, session length, and frequency. These analyses help identify specific conditions under which physical activity is effective, thereby offering exploratory guidance for clinical decision-making.

### 4.2 Influence of exercise prescription on depression in middle-aged and younger-old adults

#### 4.2.1 Exercise modalities

Among the exercise types, Tai Chi showed the largest effect, followed by yoga. Walking or running interventions did not reach statistical significance. However, heterogeneity remained high within the Tai Chi subgroup (I^2^ = 88%). This is likely due to the small number of available studies, variations in Tai Chi protocols, and differences in baseline depression severity and outcome measures across studies. Importantly, despite statistical heterogeneity, the direction and magnitude of the effect were consistently favorable in all Tai Chi studies. Thus, the finding remains clinically informative. Further subgroup analyses by intervention duration, session length, and frequency may help generate preliminary dose-related observations for Tai Chi in this population.

It should be noted that, although our subgroup analysis showed a larger effect for Tai Chi than for yoga or walking/running, this comparison is confounded by differences in intervention duration. Tai Chi interventions tended to be longer, whereas most walking/running trials were shorter. Thus, the apparent advantage of Tai Chi may partly reflect a higher total exercise dose rather than a unique effect of the exercise modality itself. To our knowledge, no eligible study directly compared different exercise types under identical dose conditions. Therefore, we cannot conclude that Tai Chi is superior to other modalities. The observed difference may partly reflect dose effects rather than modality-specific effects. Moreover, these subgroup analyses are based on indirect, study-level comparisons, which are susceptible to confounding by baseline depression severity, intervention dose, and other study-level characteristics. Therefore, any apparent advantage of Tai Chi in this analysis should be interpreted solely as hypothesis-generating, not as evidence of superiority.

Evidence has shown that resistance training may improve physical health and cognitive function (Gordon et al., 2018). However, no studies on resistance training meeting the inclusion criteria were included in this review. One possible explanation is that middle-aged and elderly individuals have low adherence to resistance training, which may limit its actual effect. Interestingly, the combination of aerobic and resistance training has been proven to help regulate mood (Wang H. et al., 2025). This may be because these studies cover a wider age range; or because the intervention of aerobic exercise may promote the release of serotonin and dopamine, thereby reducing anxiety and stress (Wang H. et al., 2025). In summary, for this population, physical activity can be regarded as a beneficial therapeutic tool rather than a source of additional stress.

Tai Chi is a mindfulness-based aerobic exercise. By improving attention, it may optimize heart rate variability (Chiang et al., 2024; Liao et al., 2024), reduce neurotoxic metabolites (Jing, 2025), restore normal response (Zhao et al., 2024) and lower fall risk. To better understand the potential pathways that might underlie the observed effects, and provide references for subsequent studies. The potential mechanisms were explored based on published experiments. Although the present meta-analysis cannot determine whether Tai Chi is superior to other exercise modalities, previous experimental studies have proposed several potential mechanisms through which Tai Chi might influence depressive symptoms. These proposed mechanisms include regulation of the hypothalamic–pituitary–adrenal (HPA) axis (Yu et al., 2018), modulation of brain-derived neurotrophic factor (BDNF) expression (Luo et al., 2024), reduction of inflammatory cytokines (Leal-Galicia et al., 2019; Luo et al., 2024), and regulation of specific microRNAs (Jin et al., 2019; Na and Kim, 2021; Cerna et al., 2024). However, none of these mechanisms were directly tested in the included trials, and they remain speculative. Future studies are needed to examine whether these pathways differ across exercise types. Together, these mechanistic hypotheses suggest that Tai Chi warrants further investigation, but do not constitute evidence of superiority (Huang et al., 2025).

Nevertheless, some evidence suggests that Tai Chi may not significantly affect depression (Kraft et al., 2024); further analysis indicates that the intervention duration might have been too short. Yoga also shows potential for improving depression and sleep quality, but its efficacy may be more beneficial for patients with chronic severe depression (West et al., 2021). Therefore, for middle-aged and younger-old adults with depression, Tai Chi may produce better intervention effects by inducing synergistic physiological and psychological responses.

#### 4.2.2 Intervention duration

The number of included RCTs was small, and the training durations varied widely. If the durations were further divided into more subgroups in our analysis (e.g., 2, 3, 4, 6, and 12 months), some subgroups would have too little evidence. This would make it difficult to assess heterogeneity and perform effective analyses. Li et al. (2024); Zhu et al. (2024); and Dong et al. (2025) all found that interventions exceeding 6 months significantly alleviated depressive symptoms. Therefore, we set 6 months as the cutoff for subgroup analysis. Only two studies examined outcomes beyond 6 months. However, the difference between the groups was significant (*p* < 0.001), pointing to a trend worth noting. Nevertheless, given the limited sample size, more long-term studies are needed to confirm this finding.

Our findings suggest that exercise interventions lasting at least 6 months were associated with larger effect estimates in the included studies. However, due to the small number of long-term studies (k = 2), this finding is preliminary. However, research by Jin (Jin et al., 2019) and Schulte (Schulte et al., 2022) demonstrates that aerobic training, regardless of duration, is effective in reducing depression and improving cognitive performance among this population. Yet, a study indicate that a minimum of 12 weeks of training is required to meaningfully reduce depression and anxiety, and to improve quality of life and aerobic capacity. Pushing this further, Zhang (Zhang et al., 2025) conducted a meta-analysis identifying 16 weeks as the most effective intervention length.

This inconsistency with our results could be explained by variations in how depression was measured or the severity levels of participants across the included trials. Notably, middle-aged and younger-old adults with depression may recover physical function and adapt more slowly. Short-term interventions might not produce significant effects in this population. Given the time-dependent nature of exercise benefits, establishing long-term regular exercise habits appears particularly important for this group. Such habits can promote brain neuroplasticity and optimize HPA axis function (Li et al., 2024).

#### 4.2.3 Single exercise duration and weekly frequency

These findings are consistent with those of Danny (Danny et al., 2023), Butler (Butler et al., 2008), Rao Ting (Rao et al., 2021), and align with the WHO guidelines recommending 150–300 min of moderate-intensity aerobic activity per week (World Health Organization, 2018). Notably, subgroup analysis of session duration revealed that both longer and shorter sessions significantly alleviated depressive symptoms. However, the effect size for shorter interventions was relatively small. These results can help guide the design of exercise programs for this population with depression.

The link between exercise and depression is well documented, yet the dosage remains debated. Zhang (Zhang et al., 2025) identified a regimen of three weekly sessions under 45 min as most effective for younger-old adults. However, findings from a larger cohort suggest that insufficient activity may be harmful. For example, Paulo (Paulo et al., 2016) reported that less than 150 min of weekly exercise was associated with elevated depression risk and cognitive decline. In terms of single exercise duration, a Mendelian randomization study by Choi (Choi et al., 2019) demonstrated that each hour of moderate activity reduced depression risk by 26%. Of course, frequency also appears critical. Chen (Chen et al., 2022) observed that exercising more than three times per week yielded significantly greater symptom relief than lower frequencies. Collectively, the above studies indicate that both single-session exercise duration and weekly exercise frequency exhibit threshold effects, significantly influencing the improvement of depression.

This study centered on middle-aged and younger-old adults with depression. This population often exhibits reduced neuroplasticity and emotional regulation capacity. As a result, their ability to engage in and adapt to exercise may be limited. Longer sessions may help regulate and sustain neuroendocrine function (Szczegielniak and Krivošová, 2025). They may also promote functional recovery in the cerebral cortex and limbic system. These changes could enhance both psychological and physical adaptation. In addition, exercise frequency also matters. Sessions held at least three times per week may strengthen neuromodulatory effects. More importantly, this frequency prevents long intervals that can diminish exercise benefits. In conclusion, the findings suggest that a regimen of at least 60-min sessions performed three or more times weekly was associated with larger effect estimates in this population. This combination was associated with larger effect estimates in the included trials in the included trials, but further confirmation is needed.

#### 4.2.4 Analysis of combination medications

To test whether the antidepressant benefits of physical activity are independent of medication, eligible RCTs were stratified according to baseline antidepressant use. Our subgroup analysis showed that physical activity alone had a significant intervention effect in this population. In contrast, exercise combined with medication did not show a statistically significant additional effect in the pooled analysis. This may reflect insufficient statistical power or heterogeneity across studies, rather than a complete absence of benefit. This finding does not contradict the overall conclusion that exercise benefits depression. It is consistent with the meta-analysis by Schuch and colleagues (Schuch et al., 2016). They found that exercise significantly improved depressive symptoms in unmedicated patients. However, among those on stable medication, adding exercise provided no additional benefit. This result may be explained by a ceiling effect (Halamová et al., 2019). Medication may already control symptoms, leaving little room for exercise to further reduce them.

Nevertheless, other studies have reported conflicting conclusions. Noetel found a moderate effect for combined medication and exercise (Noetel et al., 2024). This suggests that exercise offers measurable adjunctive benefits for medicated patients with depression. An earlier Cochrane review also supported that exercise has mild to moderate antidepressant effects, regardless of medication status (Mead et al., 2009). This implies that the effect of exercise may not depend entirely on baseline medication use. These inconsistent findings may be related to differences in depression severity or medication regimens. In addition, variations in study design and exercise protocols may further explain the heterogeneous subgroup effects observed in the current evidence.

### 4.3 Limitations

While this meta-analysis quantifies the antidepressant effects of physical activity, several notable limitations deserve elaboration. High statistical heterogeneity was detected across the nine included RCTs (I^2^ = 89%, τ^2^ = 0.72). Leave-one-out sensitivity analysis verified that no single trial dominated the pooled outcome, yet the substantial residual heterogeneity could not be fully interpreted. The 95% prediction interval of the overall effect spanned zero from −2.90 to 1.36. Although the pooled SMD reached statistical significance with its 95% confidence interval excluding zero, the wide prediction interval indicates variable true effect sizes across distinct populations, intervention schemes and outcome indicators. Crucially, this high residual heterogeneity constrains the generalizability of the pooled effect estimates.

Subgroups containing fewer than four trials such as Tai Chi interventions, interventions lasting no less than 6 months and single sessions longer than 60 min lacked sufficient degrees of freedom, leading to unstable prediction intervals that were not presented here. The limited total number of included studies precluded reliable meta-regression analyses due to insufficient statistical power, and larger pooled datasets are required for further exploration of heterogeneity origins. Noticeable clinical heterogeneity was also observed in baseline depression severity, rating scales including HRSD, BDI and GDS, as well as diverse exercise modalities, frequency, intensity and duration. Combined with statistical heterogeneity, clinical variability further narrows the external applicability of our results.

Blinding participants to exercise allocation is practically unachievable given the nature of physical activity intervention (Hird et al., 2024). Participants can readily discriminate their assigned treatment protocol, which may introduce expectation bias and placebo effects and potentially overestimate the therapeutic benefits of exercise (Hird et al., 2024). Relevant evidence confirms that such expectancy bias exerts only mild influences on self-reported mood scales (Stothart et al., 2014). Even so, assessor blinding and active control arms such as stretching or health education should be adopted in subsequent trials to isolate the specific efficacy of exercise interventions. Funnel plot assessment for publication bias was not feasible with fewer than 10 eligible studies, yet leave-one-out sensitivity analysis indirectly supported the robustness of primary results. The small total sample of trials and enrolled participants lowered statistical power and estimation precision, and rendered subgroup effect estimates unstable. Furthermore, subgroup analyses are susceptible to ecological confounding, so all subgroup outcomes must be interpreted cautiously and treated solely as exploratory hypothesis-generating findings rather than definitive conclusions.

Additionally, the operational definition of “younger-old adults” (mean age 45–65 years) used in this review is not a standard geriatric classification. This age range was chosen to reduce confounding from advanced age-related conditions, but it limits direct comparison with studies that define older adults as ≥65 years. Therefore, future studies with larger sample sizes and longer follow-up periods are needed to validate these preliminary findings and to establish more definitive exercise prescriptions.

### 4.4 Strengths of the study

This meta-analysis possesses multiple methodological strengths that improve the rigor of current evidence. Strict inclusion criteria were adopted to exclude individuals with severe comorbidities such as cancer, stroke and Alzheimer’s disease, minimizing confounding effects from additional illnesses. Unlike many previous syntheses, the present study stratified effect sizes according to antidepressant medication status, which helped isolate the independent therapeutic effect of physical activity. Beyond conventional confidence intervals, we incorporated prediction intervals and GRADE certainty evaluation to achieve more transparent assessment of heterogeneity and research reproducibility. Furthermore, medication-related information was extracted directly from original RCTs, a methodological detail frequently overlooked in prior meta-analyses. These standardized procedures strengthen the internal validity and transparency of our results, although the overall certainty of evidence remains low due to inherent study limitations.

## 5 Conclusion and future perspectives

Among the limited and exploratory findings of this study, adding exercise to pharmacotherapy was not associated with a statistically significant improvement in depression outcomes. This finding should be considered preliminary and requires replication in larger, well-powered studies. However, physical activity reduced depressive symptoms in participants who were not taking antidepressant medication. Exploratory subgroup analyses suggested that Tai Chi was associated with larger effect estimates than walking or running. Given the indirect nature of these comparisons and potential confounding by intervention dose, no conclusion about the superiority of any exercise type can be drawn. These findings should be considered hypothesis-generating.

Interventions lasting 6 months or longer, with sessions exceeding 60 min performed at least three times per week, were associated with larger reductions in depressive symptoms. However, due to the low certainty of evidence (GRADE), the small number of included studies, and the indirect nature of subgroup comparisons, these findings should be considered hypothesis-generating and require confirmation in adequately powered randomized controlled trials.

Subsequent studies ought to adopt larger-scale and rigorously designed randomized controlled trials. Participants need to be grouped according to antidepressant usage, sex, baseline depression severity and standard age brackets including 45–54 years, 55–64 years and 65 years and older. Extended intervention cycles together with blinded outcome evaluation are also required to further improve the reliability of research conclusions.

## Data Availability

The original contributions presented in the study are included in the article, further inquiries can be directed to the corresponding authors.
